# Editorial: Neurogastroenterology – Focus on the Gut-Brain Axis

**DOI:** 10.3389/fpsyt.2021.653910

**Published:** 2021-03-04

**Authors:** Andreas Stengel, Guillaume Gourcerol, Yvette Taché

**Affiliations:** ^1^Department of Psychosomatic Medicine and Psychotherapy, University Hospital Tübingen, Tübingen, Germany; ^2^Department for Psychosomatic Medicine, Charité Center for Internal Medicine and Dermatology, Charité-Universitätsmedizin Berlin, Corporate Member of Freie Universität Berlin, Berlin Institute of Health, Humboldt-Universität zu Berlin, Berlin, Germany; ^3^Rouen University Hospital - INSERM UMR 1073 / INSERM CIC-CRB 1404, Rouen, France; ^4^Vatche and Tamar Manoukian Digestive Diseases Division, Department of Medicine, UCLA/Digestive Diseases Research Core Center, UCLA David Geffen School of Medicine, Los Angeles, CA, United States; ^5^VA Greater Los Angeles Healthcare System, Los Angeles, CA, United States

**Keywords:** irritable bowel syndrome, Functional dyspepsia (FD), microbiome, psychosomatic aftercare, stress

The gut-brain axis has drawn increasing attention during the past decade as reflected by the exponential number of publications in this field. Somatoform gastrointestinal disorders such as functional dyspepsia (FD) and irritable bowel syndrome (IBS) are very common and characterized by alterations of gut-brain signaling (1, 2) that has recently been expanded to the gut microbiome referred to as microbiome-gut-brain axis (3). The importance of these pathways has been taken into consideration in the new Rome IV criteria in their naming of “disorders of the gut-brain interaction” (4). The present special issue highlights 18 original and review articles covering the broad range of investigations including the pathophysiology of functional disorders, the microbiome-gut-brain axis, and treatment options.

The pathophysiology of functional disorders is complex and multifactorial related to alterations taking place along the gut-brain axis as reflected by eight articles spanning from human colonic mucosal biopsies to brain network alterations. Two original studies performed on colonic biopsies of patients with IBS focused on brain-derived neutrophic factor (BDNF) (Konturek et al.) and mucosal Tuft cells Aigbologa et al. Building on recent reports indicative of a connection between BDNF and IBS (5), Konturek et al. showed a sex difference in BDNF mRNA assessed in colonic biopsies of patients with IBS diarrhea subtype (IBS-D) with females having a higher expression than males. This raises interesting possibilities of a role of BDNF in the recognized sex difference of IBS, considered to be female-predominant (6). Aigbologa et al. provide novel evidence that colonic biopsies of patients with non-post-infectious IBS have Tuft cell hyperplasia taking place selectively in the subgroup of IBS-D. This is paralleled by an increased secretion of Tuft cell product marker, IL-25, in biopsies and elevated plasma levels. By contrast, there is no change in patients with constipation-predominant IBS. These interesting observations open new fields of investigations as whether Tuft cell hyperplasia can represent a step forward in the identification of morphologic characteristics specific for a subset of patients with IBS-D. The comprehensive review by Mahurkar-Joshi and Chang provides emerging evidence that IBS is associated with several DNA methylation changes detected in peripheral blood mononuclear cells or colonic mucosa. The review also delineates the growing interest in the role of microRNA and long non-coding RNAs in the etiopathology of IBS. Of interest, as part of the underpinning factors in IBS, recent reports are indicative of interactions between the microbiome, diet, and epigenetic factors labeled as the “microbiota-nutrient metabolism-epigenetic axis” (Mahurkar-Joshi and Chang). At the level of the brain, a functional magnetic resonance study by Skrobisz et al. focused on the Default Mode Network in functional GI diseases. They showed changes in the left cingulum and supplementary motor area that may reflect altered processing of homeostatic stimuli. Two clinical studies, performed in healthy volunteers probed mechanisms of visceroception in response to colorectal distension stimuli to address the influence of sex or chronic stress. Labrenz et al. unraveled that healthy women had difficulties to disengage from threat in the presence of a neutral cue indicative of a role of attentional bias mechanisms underlying hypervigilance to visceral pain. Icenhour et al. established that healthy volunteers with elevated chronic stress displayed an enhanced perception and recall bias for rectal urgency pointing to memory processes in interoceptive vigilance modulated by chronic stress.

Functional disorders in the upper gut also encompass FD and gastroparesis with symptoms that can be triggered by meal ingestion known to release gut hormones (7). The systematic review on these disorders by van den Houte et al. indicates that neither somatostatin, ghrelin nor motilin blood levels differ between patients with FD and healthy volunteers although a correlation was seen between higher burden symptoms and higher peptide hormone levels. However, it is pointed out that there are several limitations in the reported studies precluding a clear knowledge as to whether gut hormones released by nutrients can play a role in the increasing symptoms of functional diseases affecting the upper gut. Lastly, of great importance to delineate the pathophysiological mechanisms of functional gastrointestinal diseases are relevant experimental models with face value and validity. The review by Accarie and Vanuytsel provides a detailed account of the various validated rodent models mainly relevant to IBS and mechanisms along the gut-brain axis contributing to IBS-like symptoms in these models.

The gut microbiome has received growing interest as a new factor involved in the pathophysiology of functional gastrointestinal disorders (8). Small intestinal bacterial overgrowth (SIBO), defined as an increase in the number of endogenous bacteria within the small bowel, is one example of gut microbiome dysbiosis. Its diagnosis relies on small bowel aspirate culture that may be difficult to run in clinical practice, or on a positive hydrogen lactulose or glucose breath test. SIBO may mimic IBS symptoms. These include diarrhea, bloating, distention, and ultimately abdominal pain and are related, among others, to impaired intestinal permeability, chronic inflammation, and decreased absorption of bile salts. In the review from Takakura and Pimentel SIBO was confirmed to be highly prevalent in IBS. Some pathogenic bacteria, including but not limited to *Enterococcus, Escherichia coli*, and *Klebsiella*, have been reported to be associated with SIBO, while *Methanobrevibacter smithii* has been shown to increase methane production mostly in IBS patients with constipation. Therapeutic management classically relies on antibiotics, while recent studies suggested that probiotics may be new therapeutic option.

Recent studies emphasize the bidirectional communication between the gut microbiome and the brain in the maintenance of hormonal and neuro-immune homeostasis (9). Alteration of this namely “microbiome-brain-gut axis” is suspected to be a new contributing factor involved in functional gastrointestinal disorders but also in neuropsychiatric disorders (9). In the systematic review from Kraeuter et al. the authors show gut microbiome is altered in psychosis syndromes as compared to healthy controls, including alterations of Proteobacteria, Firmicutes, Bacteroidetes, Fusobacteria, and Actinobacteria phyla. These changes may therefore contribute to clarify the pathophysiology of schizophrenia-spectrum disorders that remains poorly understood. Future studies, including interventional studies, will be however required to confirm causality between these gut microbiome modifications and psychosis syndromes.

Interestingly, while there is growing interest on the characterization of interaction between brain and gut microbiota in health and disease, very little information exists regarding the salivary microbiome. In a pioneering study authored by Langgartner et al. the salivary microbiome was found to be modified in healthy subjects exposed to social stressor, namely the Trier Social Stress Test (TSST). In detail, the authors showed that salivary microbial beta diversity was higher in subjects with urban upbringing in the absence of daily contact with pets as compared with those that underwent rural upbringing in the presence of daily contact with farm animals. Interestingly, this was associated with increased immune activation following acute psychosocial stressor exposure induced by the TSST. Although association does not mean causation, this study casts new light on an underexplored field and suggests potential stress-induced interactions between the brain and the salivary microbiome.

Melchior et al. showed a higher prevalence of anxiety and depression in a sample of 228 subjects diagnosed with IBS compared to healthy volunteers (*n* = 228), likely contributing to the observed lower quality of life in patients with IBS Melchior et al. Interestingly, also a higher prevalence of suspected eating disorders was observed in patients with IBS compared to healthy volunteers (Melchior et al.). Based on these data and the greatly reduced quality of life due to IBS itself, it is not surprising that psychotherapy plays an important role in the treatment regimen of IBS. Hetterich and Stengel review the existing literature on psychotherapeutic options for patients with IBS and discuss psychoeducation, self-help, cognitive behavioral therapy, psychodynamic psychotherapy, hypnotherapy, mindfulness-based therapy, and relaxation therapy as evidence-based strategies for the multimodal treatment of patients with IBS (Hetterich and Stengel).

In addition, dietary approaches play a role in the treatment of functional gastrointestinal disorders. Duboc et al. review the evidence on the role of diet in symptom generation and aggravation of patients with FD and highlight conflicting data for carbohydrates, while gluten-free diets might be useful in patients specifically reporting non-celiac gluten/wheat sensitivity (Duboc et al.). Although the low FODMAP (Fermentable, Oligo, Di-, Monosaccharides, and Polyols) diet has not been tested in patients with FD so far, it might still be useful due to the large overlap between FD and IBS (Duboc et al.), since low FODMAP has been—although being very restrictive and often aborted by the patients—shown to be effective for patients with IBS as reviewed in this special issue (Manning et al.).

Moreover, herbal therapies play a role in the management of patients with FD and IBS with STW-5, peppermint oil, rikkunshito and DA-9701 being best studied. These compounds affect different functions of the gastrointestinal tract such as inflammatory, sensory, and motor functions (Kim et al.). Although first studies showed efficacy and safety of these compounds in controlled trials, high quality studies on pharmacological mechanisms and clinical effects of these herbal drugs are largely missing (Kim et al.). Although the placebo effect should be regarded in these studies, it does not seem to be higher in patients with functional gastrointestinal disorders compared to other functional disorders– contrary to the commonly discussed opinion (Enck and Klosterhalfen). Non-response rates following various treatment attempts are high in patients with IBS ranging from 63.0 to 83.9% and likely contribute to the low quality of life observed in these patients as shown in a current original article (Dong et al.).

In summary, this special issue from Frontiers in Psychiatry—Psychosomatic Medicine highlights the role of brain-gut interactions and their disturbance in the pathophysiology of functional gastrointestinal disorders. The different research articles published in this issue add to the understanding of brain-gut complex interactions, which involve gut microbiome, diet, genetics, neuro-hormonal, and neuro-immune response. Future directions should encompass mechanistic as well as clinical interventional studies to precise the disease-specificity as well as the causal relationships of these alterations in the context of functional gastrointestinal disorders.

## Author Contributions

All authors prepared, revised, and finalized the article.

## Conflict of Interest

The authors declare that the research was conducted in the absence of any commercial or financial relationships that could be construed as a potential conflict of interest.
